# Identification and characterization of a novel small viral peptide (VSP59) encoded by *Bombyx mori* cypovirus (BmCPV) that negatively regulates viral replication

**DOI:** 10.1128/spectrum.00826-24

**Published:** 2024-10-09

**Authors:** Manman Cao, Qunnan Qiu, Xing Zhang, Wenxue Zhang, Zeen Shen, Chang Ma, Min Zhu, Jun Pan, Xingyu Tong, Guangli Cao, Chengliang Gong, Xiaolong Hu

**Affiliations:** 1 School of Life Science, Soochow University, Suzhou, China; 2 School of Chemistry and Life Science, Suzhou University of Science and Technology, Suzhou, China; 3 Institute of Agricultural Biotechnology and Ecological Research, Soochow University, Suzhou, China; David Geffen School of Medicine at UCLA, Los Angeles, California, USA

**Keywords:** BmCPV, sORF, VSP59, apoptosis, viral replication

## Abstract

*Bombyx mori* cypovirus (BmCPV), a member of the Reoviridae family, is a well-established research model for double-stranded RNA (dsRNA) viruses with segmented genomes. Despite its small genome size, the coding potential of BmCPV remains largely unexplored. In this study, we identified a novel small open reading frame within the S10 dsRNA genome, encoding a small viral peptide (VSP59) with 59 amino acid residues. Functional characterization revealed that VSP59 acts as a negative regulator of viral replication. VSP59 predominantly localizes to the cytoplasm, where it interacts with prohibitin 2 (PHB2), an inner membrane mitophagy receptor. This interaction targets mitochondria and triggers caspase 3-dependent apoptosis. Transient expression of *vsp59* in BmN cells suppressed viral replication, an effect that was reversed by silencing PHB2 expression. Moreover, recombinant BmCPV with a mutated *vsp59* exhibited reduced replication. Our findings demonstrate that VSP59 interacts with PHB2 on mitochondria, inducing apoptosis and thereby diminishing viral replication. This study expands our understanding of the genetic information encoded by the BmCPV genome and highlights the role of novel small peptides in host-virus interactions.

**IMPORTANCE:**

A novel small open reading frame (sORF) from the viral genome was identified and characterized. The sORF could encode a small viral peptide (VSP59) that targeted mitochondria and induced prohibitin 2-related apoptosis, further attenuating *Bombyx mori* cypovirus replication.

## INTRODUCTION

As a typical double-stranded RNA (dsRNA) virus, *Bombyx mori* cypovirus (BmCPV) belonged to the family Reoviridae. It is one of the most serious pathogens in sericulture. BmCPV genome is composed of 10 discrete dsRNA segments (S1–S10) (1). Each segment has generally been assumed to be monocistronic, and only the plus (+) strand of the dsRNAs has protein-coding open reading frames (ORFs) with translation activity (2). However, there has been increasing evidence in recent years that the unforeseen prevalence of unannotated small ORF (sORF) translates proteins with lengths less than 100 amino acid residues from eukaryotes, plants, bacteria, and other viruses (3–6). sORF-encoded peptides were associated with many biological processes, such as cell communication, signal transduction, transcriptional regulation, and metabolism (7).

Virus-derived small peptides are evolutionary endogenous regulatory factors that could help viruses evade immune surveillance in the hosts (8). A potential novel viral product was generated from the BmCPV segment 7 with a leaky scanning mechanism (9). Also, a novel sORF was identified from the S5 segment of BmCPV, which could negatively regulate BmCPV infection (10). These findings indicated that the 10 dsRNA segments of BmCPV encoded more than 10 proteins; the segmented genome also encoded other small proteins or peptides with unknown biogenesis. In addition, increasing evidence showed that small viral peptides were identified and characterized in DNA and RNA viruses, which may be manipulated by the hosts to adjust viral genetic decoding responding to environmental alteration (11).

On the other hand, several discovered virus-derived small peptides could inhibit virus infection in the hosts (10, 12, 13). Hitherto, very few works characterize small viral peptides from the reovirus. Here, we identified a small viral peptide (VSP59) composed of 59 amino acids from the conserved sequence of the S10 segment encoding a non-structural protein, polyhedrin (14). VSP59 targeted the mitochondria, accelerated apoptosis, and suppressed viral replication in transfected cells. We concluded that VSP59 is an evolutionary product that utilized apoptosis to balance host cell survival and viral replication. And highlights the role of novel small peptides in host-virus interactions.

## MATERIALS AND METHODS

### Cell culture, BmCPV virion preparation, and infection

BmN cell lines were cultured in the TC-100 insect medium (Sigma-Aldrich) with 10% heat-inactivated fetal bovine serum. Cells were cultured at 26°C.

BmCPV virions were purified from the BmCPV polyhedron, collected from the midguts extracted from BmCPV-infected silkworm larvae according to our previous report (15).

BmN cells were inoculated with BmCPV [multiplicity of infection (MOI) = 2] at 4°C for 30 min, followed by incubation at 26°C for 30 min. Then the cellular supernatant was discarded, and the complete insect medium was added to the cells.

### 5′-rapid amplification of cDNA ends

Silkworm midgut was harvested for total RNA extraction, and the 5′ first-strand cDNA was synthesized using the Smart RACE cDNA amplification kit (Takara, Dalian, China). The resORF10-1, resORF10-2, resORF10-3, and resORF10-4 primers for 5′ cDNA synthesis were listed in Table 1. The Adavatage 2 PCR kit (Takara, Dalian, China) was utilized for PCR, and the PCR products were cloned into the pMD19-T vector (Takara, Dalian, China) for Sanger sequencing.

**TABLE 1:** Primers used in this study

Primers	Sequences	Detected genes
S10-sORF1	GGATCCATGTACGCACCATCAAAATG	S10-ORF*-Bam*HI/*Sac*II
S10-sORF2	GAGCTCTTGCGAAATGGAGCTATATTC	
BmCPV10-2	CGTCTAGACCGGTATCCAAGTTACACGAGC	BmCPV S10
BmCPV10-1	GCGAATTCGGATCATGGCAGACGTAGCAGG	
resORF10-1	GGTAACTCACTCTGTACC	S10-sORF
resORF10-2	CCAACGATTTCTCCGTCTC	
resORF10-3	CGCATACTACTCAGATGGG	BmCPV S10
resORF10-4	CGTTGTGTTGTCCCTCGCG	
BmSOCS2-1	GTGACAGACCGTTGGCTAGG	BmSOCS2
BmSOCS2-2	GCACCGGCGAGTGTGGACAC	
Bmstat-1	GAGCGTTATGGACGAGAAGC	Bmstat
Bmstat-2	CCTGGTTGCCGTGGACTATG	
BmPGRP-LE-1	CACTGCAACAGAAAGCTGTAG	BmPGRP-LE
BmPGRP-LE-2	CGCAATATGCCGATCCGTCAC	
Bmdrc2-1	CCAGCGTTCACGTCCGTTGGA	Bmdrc2
Bmdrc2-2	GATCGCCAAGATTTGGTCGAA	
S10-sORF-T7	TAATACGACTCACTATAGGTACGCACCATCAAAATGGC	T7-S10-sORF
S10-sORF2	GAGCTCTTGCGAAATGGAGCTATATTC	
GFP-1T7	TAATACGACTCACTATAGGATGGCTAGCAAAGGAGAAG	T7-GFP
GFP-2T7	TTGTGCCCATTAACATCACC	

### Plasmid construction, transient expression, and real-time PCR

**Fig 1:**
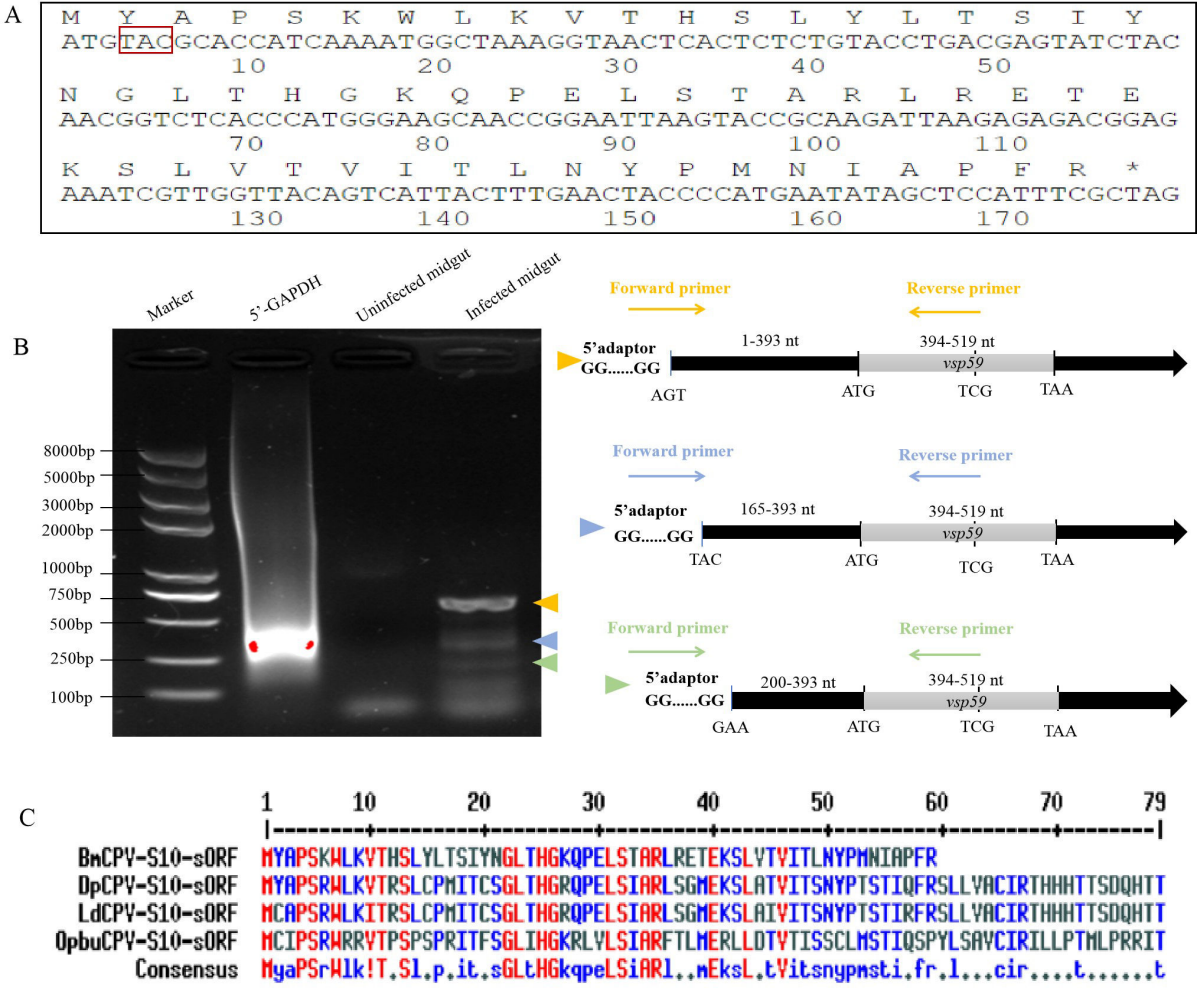
Sequence of a novel viral small peptide VSP59 expressed in BmCPV-infected midgut. (**A**) The nucleotide sequence of vsp59 and the predicted amino acid sequence. The sequence in the red frame would be mutated to construct pIZT-vsp59^mut^. (**B**) 5′-rapid amplification of cDNA ends (RACE) for characterization of previously unknown 5′ cDNA sequence of VSP59 sORF. The diagram of the novel transcripts was identified by 5′ RACE. Colored arrowheads indicate bands with specific RACE products and match colors in the right diagrammatic sketch. (**C**) Amino acid sequence alignment of VSP59 against other predicted amino acid sequences from *Dendrolimus punctatus* CPV (DpCPV), *Lymantria dispar* CPV (LdCPV), and *Operophtera brumata* CPV (ObCPV). The same red word indicates identical amino acids in all four sequences.

The *vsp59* gene was cloned with PCR using primer pairs S10-sORF1/S10-sORF2 (Table 1). Then the *vsp59* gene sequence was cloned into the *Bam*HI/*Sac*I sites of pET28a (+) vector (Novagen, Darmstadt, Germany) to generate recombinant plasmid pET28a-vsp59. The complete ORF of *vsp59* gene was inserted into pIZT-V5/His vector (Invitrogen, Frederick, USA) to obtain the recombinant plasmid pIZT-vsp59. The *vsp59* gene with the second codon TAC (red frame in Fig. 1A) mutated to the terminal codon TAA was cloned into pIZT-V5/His to obtain a recombinant plasmid pIZT-vsp59^mut^. The ORF of vsp59 in the complete S10 fragment was replaced with the red fluorescent protein (dsRed) gene, and then it was cloned into pIZT-V5/His vector for the construction of pIZT-CS10-sORF-dsRed.

The recombinant plasmids were transfected into BmN cells using Lipofectamine 2000 (Thermo Fisher Scientific, Massachusetts, USA) for transient expression. At 48 h post-transfection, the cells were collected for total RNAs extraction, and the extracted RNA was reverse transcribed using TransScript One-Step gDNA Removal and cDNA Synthesis SuperMix (TransGen Biotech). Real-time PCR was carried out using the specific primers (Table 1). This experiment was repeated three times. The relative expression level of genes was calculated according to the 2^−ΔΔCt^ method (16).

### Polyclonal antibody preparation

Recombinant plasmid pET28-vsp59 was transformed into *Escherichia coli* strain BL21, and the transformed *E. coli* was induced with isopropyl-β-d-thiogalactoside at a final concentration of 1 mmol/L for 4 h at 37℃. The induced bacteria were collected, and the total proteins were extracted for sodium dodecyl-sulfate polyacrylamide gel electrophoresis (SDS-PAGE) separation and Western blotting. The His-tagged VSP59 was puriﬁed using Ni^2+^-nitrilotriacetate (Ni-NTA) resin (Invitrogen, Carlsbad, CA, USA) (17). Kunming mice (Soochow University, Suzhou, China) were immunized with the purified protein four times to prepare the antiserum according to the previous study (18). The specificity of a polyclonal antibody of VSP59 was confirmed by Western blotting.

### Western blotting

At 48 h post-transfection or at the indicated treatment times, BmN cells were harvested and washed with ice-cold phosphate-buffered saline (PBS) and lysed with lysis buffer (20 mM HEPES, 150 mM NaCl, 1 mM EDTA, 1 mM EGTA, and 1% Triton-100, pH 7.5) supplemented with protease inhibitor cocktail (Roche, Basel, Switzerland). The protein concentration in each sample was determined using a Bradford assay kit (Bio-Rad, Hercules, CA, USA). Twenty micrograms extracted protein was separated by 12% SDS-PAGE gels, followed by transfer onto polyvinylidene fluoride (PVDF) membranes (Life Technologies, Agarwal City, India). The primary antibodies were anti-dsRed (Thermo Fisher Scientific, Massachusetts, USA), anti-VSP59, anti-Polh, anti-active caspase 3 (Affinity, Melbourne, Germany), anti-prohibitin 2 (PHB2) (Proteintech, Chicago, USA), anti-VP7(9) and anti-VP4 (19), and the secondary antibodies were horseradish peroxidase (HRP)-labeled goat anti-rabbit IgG (Servicebio, Wuhan, China) or HRP-labeled goat anti-mouse IgG (Servicebio, Wuhan, China). The alpha tubulin (Proteintech, Chicago, USA) served as a control. This experiment was repeated at least three times. The Western blotting image was analyzed by grayscale scanning with ImageJ software (https://imagej.en.softonic.m/?ex=BB-682.0#) .

### siRNA

BmN cells (1 × 10^6^) were transfected with 2 µg of PHB2-siRNA-245, PHB2-siRNA-637, PHB2-siRNA-750, or NC-siRNA (Table 2) which was synthesized by Integrated Biotech Solutions Corporation (Shanghai, China). The gene expression levels were determined by real-time PCR with *TIF-4A* gene as a control.

**TABLE 2:** Sequence of siRNAs

siRNA	Sense (5′–3′)	Anti-sense (5′–3′)
PHB2-siRNA-245	CAUCGUGCCAUUAUGUUCCAACAGAATT	UUCUGUUGAACAUAAUGGCACGAUGTT
PHB2-siRNA-637	CCUUGAUGAUGUAUCCCUCACUGAATT	UUCAGUGAGGGAUACAUCAUCAAGGTT
PHB2-siRNA-750	GGGCAAAGCAAGAGCG UCAACAGAATT	UUCUGUUGACGCUCUUGCUUUGCCCTT
NC-siRNA	UUCUCCGAACGUGUCACGUTT	ACGUGACACGUUCGGAGAATT

### Immunoprecipitation

The supernatant from lysed BmCPV-infected BmN cells or midguts was incubated with anti-VSP59 or pre-immune serum (as a control) overnight at 4°C, followed by immunoprecipitation with Protein A/G agarose beads (Sangon, Shanghai, China). The immunoprecipitation complex was washed three times with lysis buffer. Then the complex was subjected to SDS-PAGE. The protein bands were visualized by silver staining. The recovered differential protein bands were used for liquid chromatograph mass spectrometer/mass spectrometer (LC-MS/MS) identification (Oebiotech Co., Ltd, Shanghai, China).

### Co-immunoprecipitation

BmN cells (1 × 10^7^) were infected with BmCPV (MOI = 2). Cells harvested at 48 h post-infection (hpi) were lysed by 120 µL NP-40 lysis buffer at 4°C for 30 min, and the supernatant was incubated with anti-VSP59 or anti-PHB2 (Proteintech, USA) overnight at 4°C, followed by incubation with 30 µL of Protein A + G Agarose (Sangon, Shanghai, China) at 4°C for 4 h. The immunoprecipitates were washed six times using pre-cold PBS. The obtained immunoprecipitates were used for Western blotting. The VSP59 and PHB2 were detected by the corresponding antibody.

### Immunofluorescence analysis

BmN cells (1 × 10^4^) were cultured in the 24 cell plate for 24 h, followed by incubation with BmCPV (MOI = 2) for 48 h. The culture medium in each well was replaced by MitoTracker Deep Red FM (Yeasen, China) diluted in culture medium (final concentration of 100 nmol/L) for continuing incubation at 26°C for 15–45 min. Briefly, BmCPV-infected cells or infected midguts were ﬁxed with 500 µL of 4% paraformaldehyde for 15 min, followed by permeabilization with phosphate buffered solution containing 1% Trixon-100 (PBST) for 5 min. Cells or midguts were incubated with a blocking buffer [5% bovine serum albumin (BSA)] for 2 h. Subsequently, cells or midguts were stained with anti-VSP59 (mouse, 1:100) and PHB2 antibody (rabbit, 1:100) as primary antibodies. Cy3 conjugated goat anti-rabbit IgG (red, 1:500) and FITC-conjugated goat anti-mouse IgG (green, 1:500) were used as secondary antibodies. The nucleus was stained with 1:1,000 diluted DAPI (blue). Images were acquired using a fluorescence microscope (TE2000U, Nikon).

### Cell cycle detection

Four micrograms of pIZT-vsp59, pIZT-V5/His, and pIZT-vsp59^mut^ vectors were transfected into BmN cells (1 × 10^6^). Forty-eight hours post-transfection, the transformed cells were harvested and fixed in the 1 mL of 70% ethanol for 3 h at 4°C, then washed the cells with 1× PBS, stained with 0.5 mL propidium iodide (PI, 50 µg/mL) in the dark for 30 min at 37°C. Flow cytometry (FC500, Beckman Coulter) was applied to detect at the 488 nm.

### Mutant virus construction

To construct a recombinant virus rBmCPV-VSP59null with missing VSP59, the second to third codon “tacgca” of VSP59 was mutated into “taagaa” in the pIZT-CS10 plasmid (20) containing a full cDNA sequence of BmCPV S10 segment, resulting in a recombinant plasmid pIZT-S10-sORF^mut^ without altering the amino acid sequence encoded by the polyhedrin gene. pIZT-CS1–pIZT-CS9 plasmids containing a full cDNA sequence of BmCPV S1-S9 segments, respectively, were constructed in our previous study (20). Then 10 recombinant plasmids (pIZT-CS1–pIZT-CS10) were digested with *Sac*II (Takara, Dalian, China) and were used as templates to produce RNAs via T7 reverse transcript kit (Takara, Dalian, China). The obtained 10 RNAs were transfected into BmN cells for the construction of recombinant virus rBmCPV. Meanwhile, replacing pIZT-CS10 with pIZT-S10-sORF^mut^, the recombinant virus rBmCPV-VSP59null was constructed. The details of the production of the recombinant virus with a reverse genetic system were referenced in our previous study (20).

### Apoptosis detection

Apoptosis was examined using an Annexin V-PE/7-AAD detection kite (10010-09; Southern Biotech) according to the procedure. Briefly, 4 µg of pIZT-vsp59, pIZT-V5/His, and pIZT-vsp59^mut^ vectors were transfected into BmN cells (1 × 10^6^). Forty-eight hours post-transfection, the transformed cells were harvested and suspended in the 1× binding buffer at a concentration of 1 × 10^6^ cells/mL. Subsequently, 100 µL of suspended cells was stained with 5 µL Annexin V/PE and 10 µL 7-AAD (20 µg/mL) for 5 min in the dark. These samples were analyzed with a flow cytometry (FC500, Beckman Coulter). Apoptotic cells in transfected cells were measured by using a TUNEL (TdT-mediated dUTP Nick-End Labeling) kit (Beyotime, China).

### Statistical analysis

Data analyses were performed with SPSS software ver. 20.0 (SPSS, Inc., Chicago, IL, USA), GraphPad Prism 8.0 (GraphPad Software, LaJolla, CA, USA) and ImageJ. All experimental data are expressed as the mean ± s.e.m. Paired *t*-test was used for statistical analysis: *P < 0.05, **P < 0.01, ***P < 0.01.

## RESULTS

### The sequence of a novel viral small peptide expressed in BmCPV-infected midguts

In our previous work (21), an RNA transcript mapping to the plus strand of genomic dsRNA segment S10 was detected by RNA-sequencing analysis in BmCPV-infected midgut. This RNA transcript was aligned to the S10 dsRNA genome reported in GenBank (accession number GQ924589), mapped to the position of 394-573 nucleotides (nt) with a predicted translation production of 59 amino acids (aa) residues. This RNA transcript had a complete sORF from the ATG to TAG, and it was shared by the transcript of polyhedrin translated by S10 (Fig. 1A). To further validate the real existence of this sORF, 5′-rapid amplification of cDNA ends was applied to characterize previously unknown 5′ cDNA sequence of sORF. Three viral RNA transcripts with a full sORF from the S10 dsRNA genome were detected (Fig. 1B).

The predicted translation product of this RNA transcript aligned to the NCBI proteins database showed high conservation at the amino acid level among *Dendrolimus punctatus* CPV (DpCPV), *Lymantria dispar* CPV (LdCPV), and *Operophtera brumata* CPV (ObCPV) (Fig. 1C). Any similar proteins/peptides genes were not identified from the other viruses with Blast analysis. Therefore, we believe it is a BmCPV-specific gene. Because the product encoded by this gene is a peptide containing 59 amino acid residues, it was named viral small peptide gene 59 (vsp59).

### A small peptide encoded by vsp59

**Fig 2:**
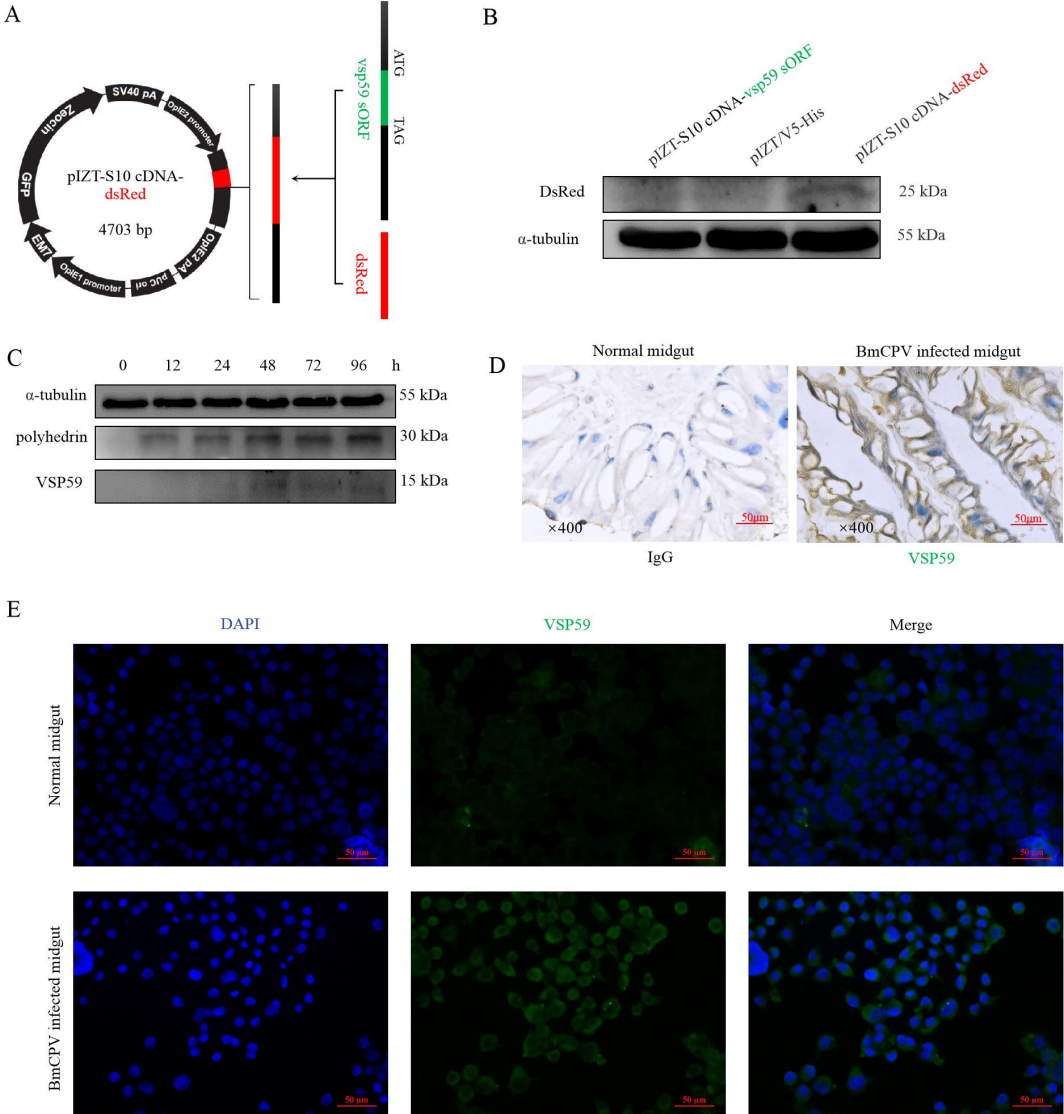
vsp59 encodes a small peptide. (**A**) Construction of pIZT-V5/His-S10 cDNA-dsRed plasmid. The dsRed gene replaced the putative sORF in the S10 cDNA sequence and was cloned into an insect-expressing vector pIZT-V5/His. (**B**) The translation activity of vsp59 was validated by Western blotting of the BmN cells transfected with the pIZT-V5/His-S10 cDNA-dsRed plasmid. The BmN cells transfected with pIZT-V5/His-S10 cDNA and pIZT-V5/His were used as controls. The primary antibody was a monoclonal antibody (mAb) against dsRed. (**C**) The expression profiling of VSP59 and polyhedrin in the infected BmN with BmCPV. The cells were harvested at 0, 12, 24, 48, 72, and 96 hpi. The total proteins extracted from these cells were analyzed by Western blotting using antibodies of anti-VSP59 and anti-Polh. α-tubulin was used as the internal reference. (**D**) Immunohistochemical detection of VSP59 in the midgut of BmCPV-infected silkworm. Immunohistochemistry was used to determine the expression levels of VSP59 protein in the enterocytes of third-instar silkworms infected with BmCPV for 96 h, with brown-yellow representing the VSP59 protein and blue representing the nucleus. Normal silkworm midgut was used as a control. (**E**) Immunofluorescence detection of VSP59 in BmCPV-infected BmN cells. BmN cells (1 × 10^4^) were infected with BmCPV for 48 h, and BmN cells (1 × 10^4^) without infection were used as a control. The primary antibody was a VSP59 antibody at 1:10 dilution, the secondary antibody was a fluorescein Isothiocyanate (FITC)-conjugated goat anti-mouse antibody (green) at 1:50 dilution, and the nucleus was stained with 4',6-diamidino-2-phenylindole (DAPI) at 1:1,000 dilution (blue) .

To address the translation activity of *vsp59*, the complete S10 cDNA sequence and the mutated S10 cDNA sequence in which the putative sORF was replaced with the dsRed gene were cloned into an insect expression vector pIZT-V5/His vector (Fig. 2A). The BmN cells were transfected with resulting constructs (pIZT-V5/His-S10 cDNA and pIZT-V5/His-S10 cDNA-dsRed) and analyzed with Western blotting or visualized by a fluorescence microscope at 48 h post-transfection. Red ﬂuorescence was observed in pIZT-V5/His-S10 cDNA-dsRed transfected cells (Fig. S1), and a signal band (25 kDa) representing the dsRed protein was identified in the pIZT-V5/His-S10 cDNA-dsRed transfected cells by Western blotting with a monoclonal antibody (mAb) against dsRed (Fig. 2B). A polyclonal antibody against VSP59 was generated by immunizing mice with recombinant protein VSP59 expressed by the transformed *E. coli* (DH5α) with pET28-vsp59 vector (Fig. S2).

10^5^ BmN cells/well were seeded in a six-well cell culture plate and then infected with BmCPV (MOI = 2). The cells were harvested at 0, 12, 24, 48, 72, and 96 h post-infection (hpi) for Western blotting. A signal band with 15 kDa was detected in the BmCPV-infected cells from the 48 hpi, and its expression level was lower than that of polyhedrin (Fig. 2C). Moreover, the silkworm was orally infected with 10^5^ polyhedra/mL coating on the mulberry leaves, VSP59 could be detected in the midguts from the infected silkworm at 96 hpi by immunohistochemistry, while the same signals were not found in the midguts from the normal silkworm (Fig. 2D). In addition, the location of VSP59 in the BmCPV-infected cells was detected with immunofluorescence staining, and the signals were mainly shown in the cytoplasm (Fig. 2E). The polyhedron was extracted from the BmCPV-infected midgut, and the virions were released with the lysis buffer (pH > 10.5). The solution of virions was used to extract viral total proteins, and the Western blotting assay showed that VSP59 could be detected from the virions (Fig. S3). These results indicated that VSP59 was actual existence.

To further verify VSP59 encoded by the BmCPV genomic RNA S10 segment, the pIZT-V5/His (a negative control), pIZT-CS10, and pIZT-S10-sORF^mut^ plasmids were transfected into BmN cells (1 × 10^6^). The total proteins extracted from the transfected cells at 48 h post-transfection were used for SDS-PAGE and Western blotting with specific anti-Polh, anti-VSP59 antibodies. Polh and VSP59 could be detected simultaneously in the cells transfected with pIZT-CS10, but VSP59 could not be detected in the cells transfected with pIZT-S10-sORF^mut^, confirming that VSP59 was encoded by the BmCPV genomic RNA S10 segment (Fig. S4A).

### The apoptosis was induced by VSP59 targeting mitochondria

**Fig 3:**
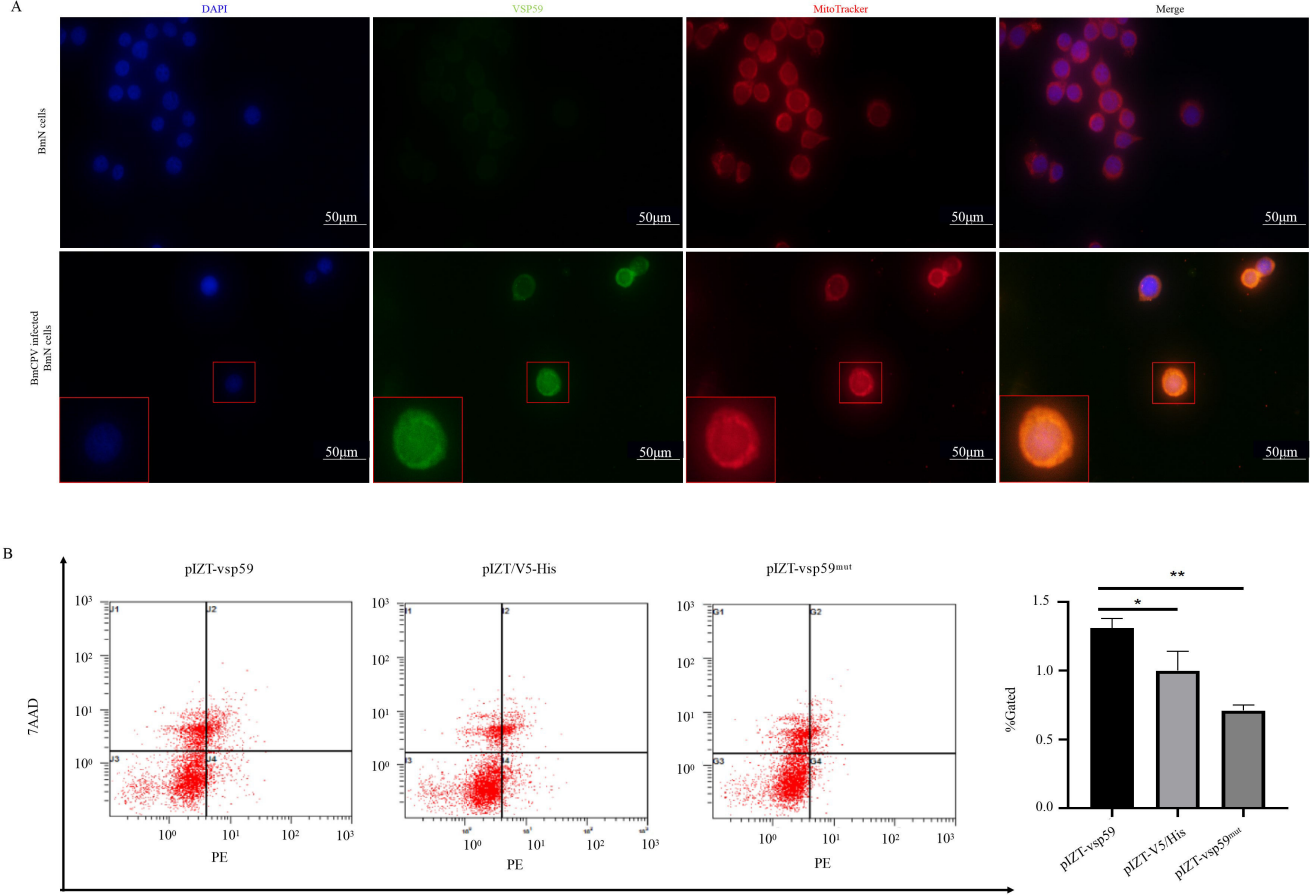
VSP59 targets mitochondria and induces apoptosis. (**A**) VSP59 co-localized with the mitochondrial membranes. BmN cells (1 × 10^4^) were infected with BmCPV (MOI = 2), and infected cells were stained with the ﬂuorescent marker MitoTracker for visualizing the co-localization with mitochondrial membranes. (**B**) VSP59 induces apoptosis. BmN cells (1 × 10^6^) were transfected with pIZT-V5/His (a negative control), pIZT-vsp59, and pIZT-vsp59^mut^ plasmids. At 48 h post-transfection, the cells were stained with Annexin V-PE/7-AAD, and the proportion of cells in different quadrants was detected by flow cytometry analysis. *P < 0.05 and **P < 0.01.

The VSP59 is localized in the cytoplasm. However, the exact roles were still vague. Confocal microscopy was applied to explore its precise subcellular localization and target organelles. BmN cells (1 × 10^4^) were infected with BmCPV (MOI = 2), and infected cells were stained with the ﬂuorescent marker of MitoTracker for visualizing the co-localization with mitochondrial membranes. As shown in Fig. 3A, VSP59 co-localized with the MitoTracker stain in the BmCPV-infected cells, suggesting that VSP59 may co-localize with the mitochondrial membranes. Accordingly, we hypothesized that VSP59 might be associated with apoptosis. To examine this hypothesis and to understand whether this event happened in cells, BmN cells (1 × 10^6^) were transfected with pIZT-V5/His (a negative control), pIZT-vsp59, and pIZT-vsp59^mut^ plasmids. Forty-eight hours post-transfection, the cells were stained with Annexin V/PE and 7-AAD, and the proportion of cells in different quadrants was detected by flow cytometry analysis. As shown in Fig. 3B, the rate of apoptotic cells was increased, and the rate of cells in the S and G2 phases was reduced in transfected cells with pIZT-vsp59 compared with pIZT-V5/His and pIZT-vsp59^mut^ (Fig. S5A). These observations showed that VSP59 might induce apoptosis in BmN cells. Apoptosis was also visible in the above-treated cells by the terminal deoxynucleotidyl transferase-mediated dUTP-biotin nick end labeling assay (Fig. S5B).

**Fig 4:**
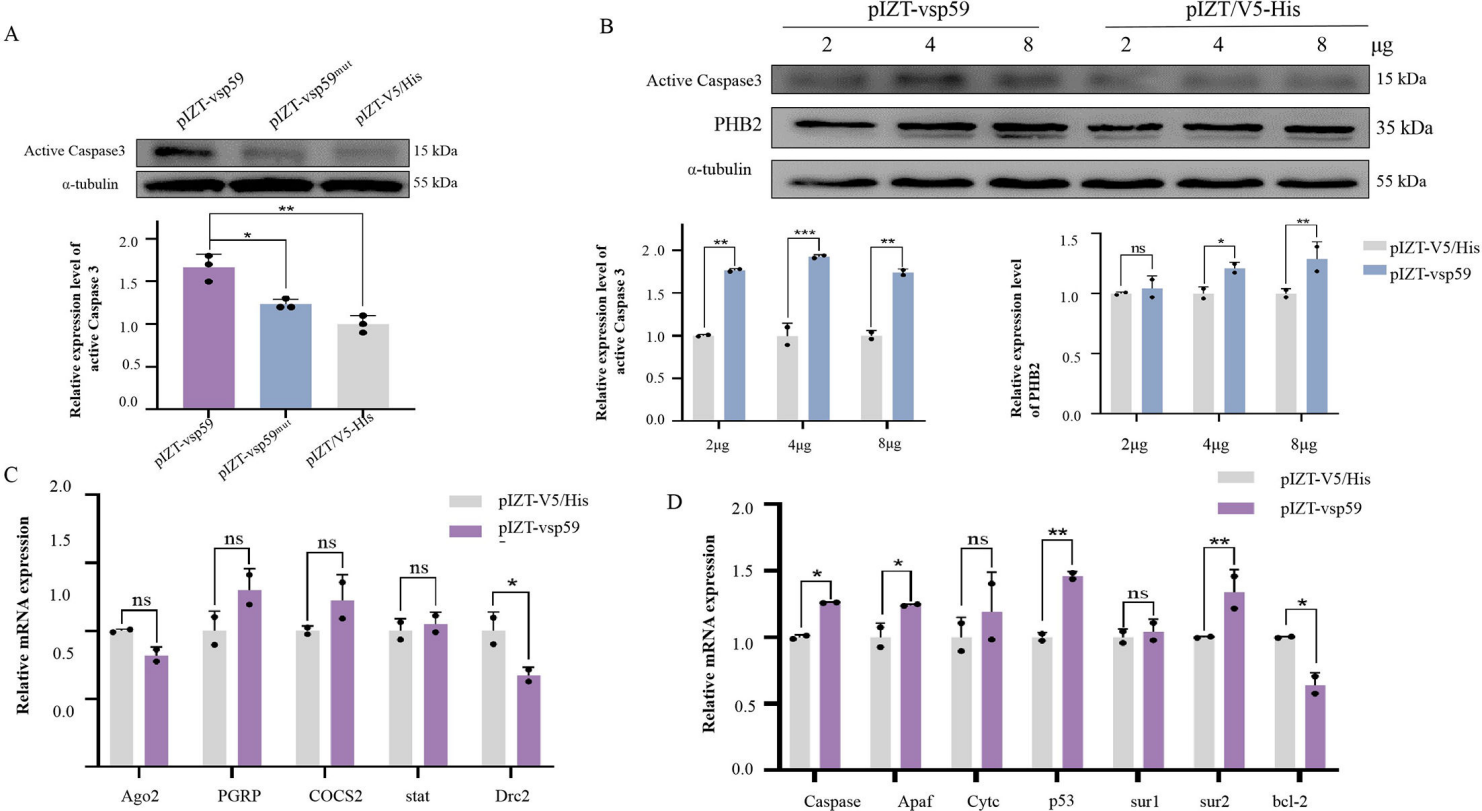
The effects of overexpression of vsp59 on the gene expression level. (**A**) The expression level of active caspase3 in the BmN cells transfected pIZT-V5/His, pIZT-vsp59, and pIZT-vsp59^mut^. The BmN cells were transfected with 4 µg of plasmids. The expression level of active caspase3 in the transfected cells was determined by Western blotting at 48 h post-transfection. Upper, Western blotting; under, grayscale scanning analysis of Western blotting signal bands. (**B**) The expression level of active caspase3 and PHB2 was increased in a VSP59-dependent manner. The BmN cells (1 × 10^6^) were transfected with 2, 4, and 8 µg of pIZT-vsp59, and the cells were collected at 48 h post-transfection to determine the expression level of active caspase3 and PHB2. The cells transfected with pIZT-V5/His were used as controls. Upper, Western blotting; below left, grayscale scanning analysis of active caspase3 signal bands; below right, grayscale scanning analysis of PHB2 signal bands. (**C** and **D**) The effects of vsp59 overexpression on the expression levels of the genes related to innate immunity signaling pathways (C) and apoptosis signaling pathway (**D**). The BmN cells were transfected with 4 µg of pIZT-vsp59. The expression level of active caspase3 in the transfected cells was determined by RT-qPCR at 48 h post-transfection. The BmN cells transfected with pIZT-V5/His were used as control. *P < 0.05, **P < 0.01, ***P < 0.001, and ns: *P* > 0.05.

To further validate apoptosis induced by VSP59, the protein expression level of active caspase3 was detected from the above three constructs transfected cells, and the results showed that the protein expression level of active caspase3 in pIZT-vsp59 transfected cells was significantly higher than that in the pIZT-V5/His /pIZT-vsp59^mut^ transfected cells (Fig. 4A). Moreover, different doses of pIZT-vsp59 were transfected into BmN cells, and the expression level of active caspase3 was detected by Western blotting. The results showed that the expression of active caspase3 and PHB2 increased in a dose-dependent manner (Fig. 4B). Meanwhile, *Caspase*, *Apaf*, *p53,* and *Sur2* genes related to apoptosis signaling pathways were significantly induced (Fig. 4C), while other immune genes related to innate immunity pathways were not changed by overexpression of vsp59 in BmN cells (Fig. 4D). Collectively, VSP59 specifically targets mitochondria and induces apoptosis.

### The interaction of VSP59 with PHB2 mediates apoptosis

**Fig 5:**
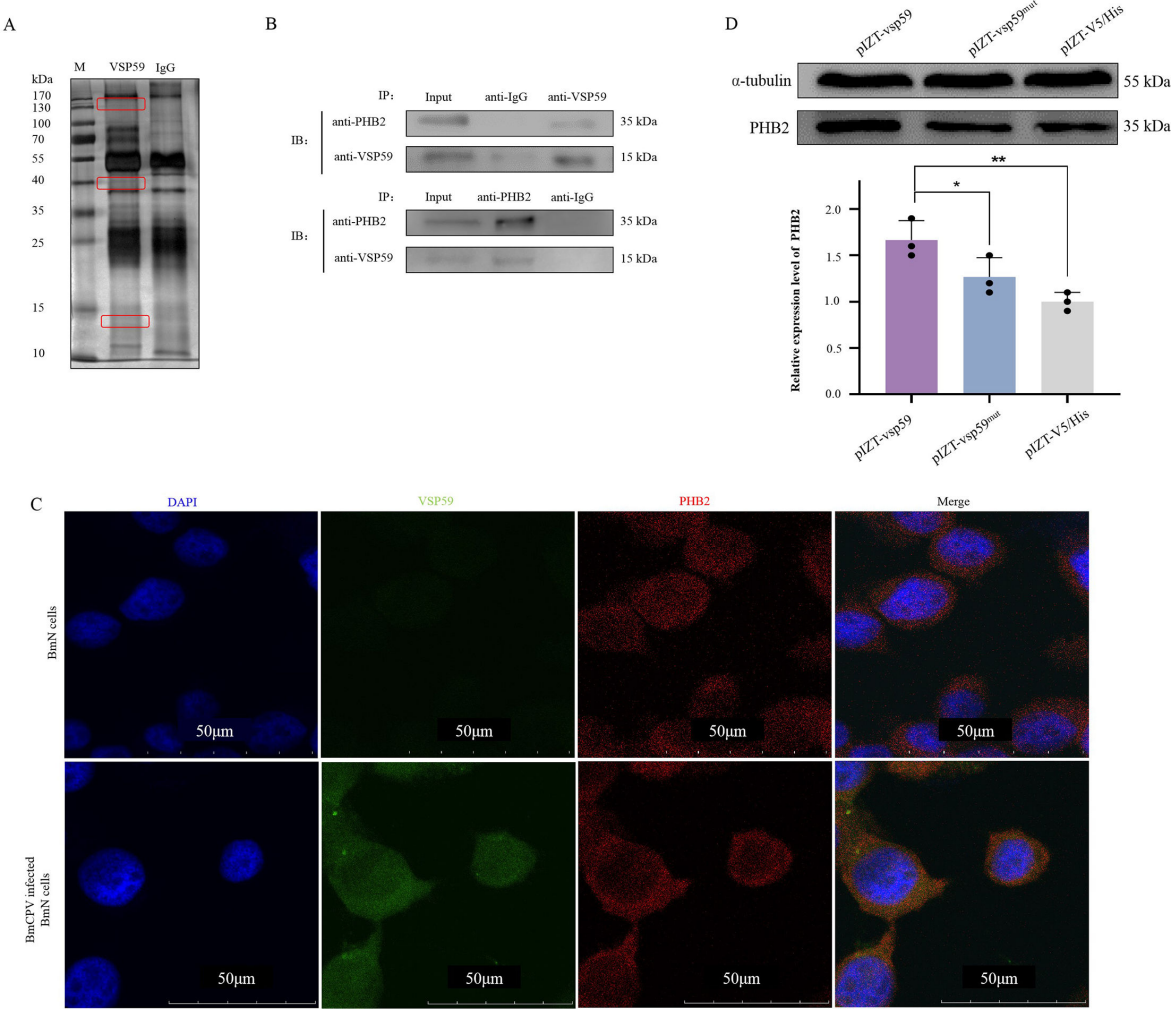
VSP59 induced apoptosis through interaction with PHB2. (**A**) Screening candidate proteins interacting with VSP59 through IP. The total proteins extracted from the infected BmN cells at 48 h post-infection were used for IP with specific anti-VSP59 and pre-immune IgG, and the generated IP complex was analyzed by SDS-PAGE. (**B**) Co-IP assay. BmN cells (1 × 10^7^) were infected with BmCPV (MOI = 2), and 48 h later, the cells were collected and lysed for Co-IP. Co-IP was performed with 5 µg of anti-PHB2, anti-vsp59, and pre-immune IgG. The obtained Co-IP complexes were separated by SDS-PAGE, and Western blotting was performed using anti-vsp59 and anti-PHB2. (**C**) The analysis of co-localization between VSP59 (green) and PHB2 (red) in BmCPV-infected cells. (**D**) The expression level of PHB2 was increased by overexpression of vsp59. The BmN cells (1 × 10^6^) were transfected with 4 µg of pIZT-vsp59. The expression level of PHB2 in the transfected cells was determined by Western blotting at 48 h post-transfection. The BmN cells transfected with the pIZT-V5/His and pIZT-vsp59^mut^ were used as control. Upper, Western blotting; under, grayscale scanning analysis of Western blotting signal bands. *P < 0.05, **P < 0.01, and ns: *P* > 0.05.

To further explore why VSP59 can induce apoptosis in the cells, immunoprecipitation (IP) was applied to screen the candidate interacted proteins from the BmCPV-infected cells. At 48 hpi, the total proteins extracted from the infected cells were used for IP and Western blotting with specific anti-VSP59 antibodies. As shown in Fig. 5A, the mass spectrometry (MS) identification results of immunoprecipitation complex showed that the peptide segments representing VSP59 could be detected in BmCPV-infected midguts (Fig. S4B). This result further confirmed the BmCPV encoding VSP59. Moreover, the peptide segments representing PHB2 were also identified (Table 3), suggesting PHB2 was a candidate protein interacting with VSP59. The physical interaction between VSP59 and PHB2 was further validated by co-immunoprecipitation (Co-IP) assay (Fig. 5B). The results of the cellular immunofluorescence assay revealed that VSP59 (green) co-localized with PHB2 (red) in BmCPV-infected cells (Fig. 5C). In addition, the expression level of PHB2 increased in the transfected cells with pIZT-vsp59 compared with the transfection of pIZT-V5/His and pIZT-vsp59^mut^ plasmids (Fig. 5D). The expression of PHB2 was found to be increased in a dose-dependent manner with pIZT-vsp59 transfection (Fig. 4B).

**TABLE 3:** Mass spectrometry identification of the interacting protein by co-immunoprecipitation

No.	Protein name	MW (kDa)/PI	Pepcount/unique pepcount	Cover percent	Sequence
1	Mitochondrial prohibitin complex protein	14/12	33176.03/9.7	35.79%	K.AM*GM*NPGYLK.L
2	Y-box protein	15/11	29332.39/11.1	42.65%	K.APQPQPQQQLEQQPQAQQAK.T
3	Elongation factor 1-alpha	11/8	50387.53/9.21	14.69%	K.EVSSYIK.K
4	Phosphate transport protein	9/8	39210.37/9.01	16.20%	K.FACFER.T
5	Eukaryotic translation initiation factor 3 subunit J	8/7	28023.82/4.85	30.71%	K.ESWEDEEEEK.K
6	RNA binding protein	7/6	26317.75/4.94	24.35%	K.FAEAQEIVR.T
7	Receptor for activated protein kinase C	6/5	36040.27/8.07	13.38%	K.LWNTLAECK.Y
8	Transcription elongation factor S-II	4/4	32552.09/9.07	15.97%	K.EAIDDAQLATVQGTK.T
9	Inhibitor of growth protein	2/2	29814.35/6.97	5.79%	K.ETDLSSVK.E
10	Apoptotic chromatin condensation inducer-like protein	1/1	24445.36/9.38	5.29%	K.YETEDQAVETR.H
11	Replication factor C (Activator 1) 5	1/1	37288.77/6.91	1.80%	R.GIGIVR.G
12	Endonuclease-reverse transcriptase	1/1	112849.4/9.59	0.61%	K.IAVPHR.K
13	Elongation factor Tu	1/1	51650.37/7.64	2.34%	K.SPEIGADAITK.L
14	Translation initiation factor 1a	1/1	17351.2/5.53	4.00%	R.KNIWVK.R

### VSP59 attenuated BmCPV replication by PHB2-related apoptosis

**Fig 6:**
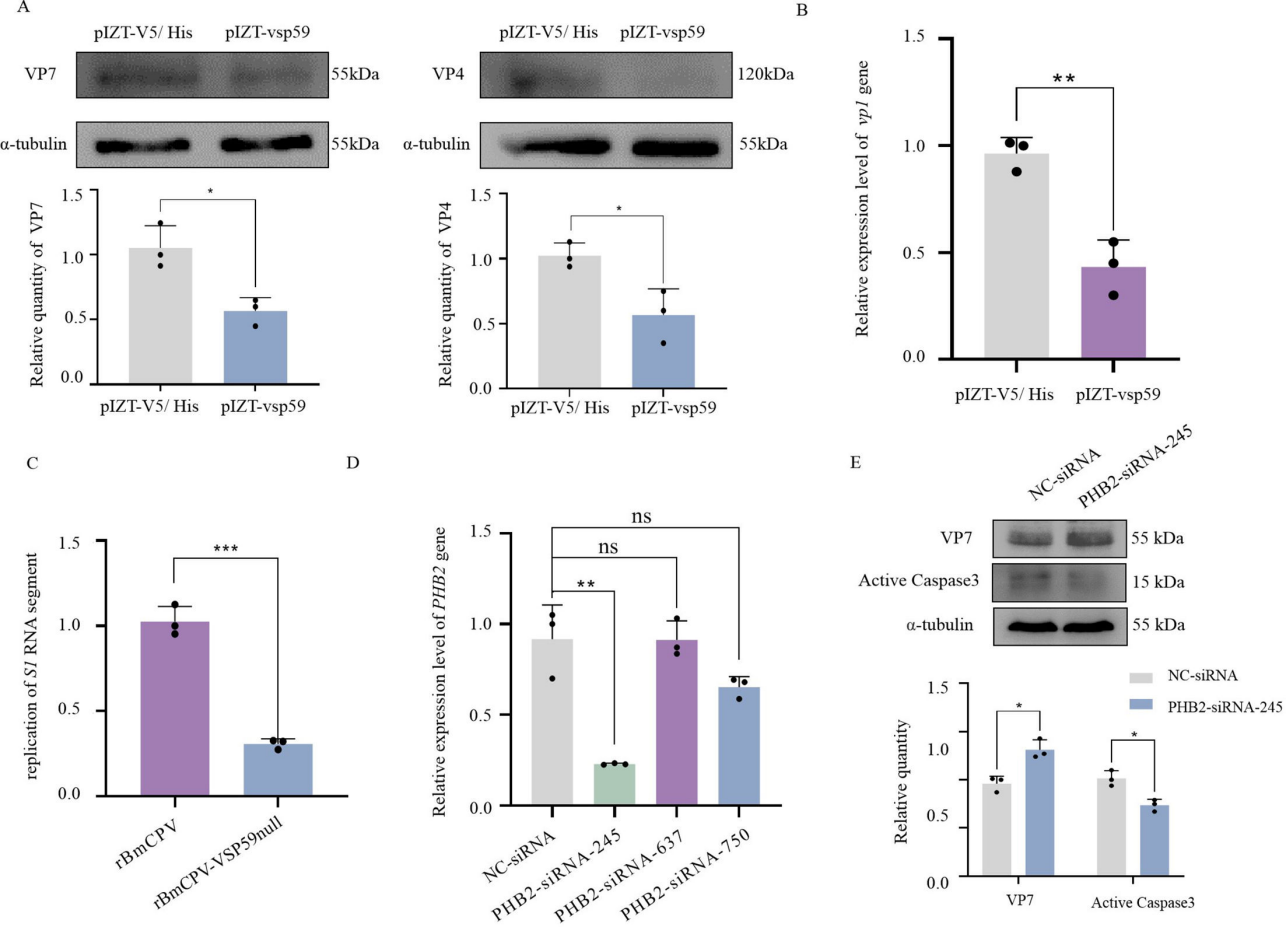
VSP59 attenuated BmCPV replication by induction of apoptosis through interaction with PHB2. (**A**) The replication of BmCPV in the transfected cells with pIZT-vsp59 was assessed by Western blotting and (**B**) real-time PCR. BmN cells (1 × 10^6^) were transfected with 4 µg of pIZT-vsp59 vector and pIZT-V5/His vector (as control). At 48 h post-transfection, the cells were inoculated with BmCPV (MOI = 2). At 48 hpi, the cells were collected. The total proteins extracted were used for Western blotting (**A**) to determine the expression levels of VP7 and VP4 viral proteins. Upper, Western blotting; under, grayscale scanning analysis of Western blotting signal bands. The total RNAs extracted were used for RT-qPCR (**B**) to determine the *vp1* gene expression level. (**C**) The recombinant BmCPV (rBmCPV) and vsp59 mutant virus rBmCPV-VSP59null were constructed using a reverse genetic system. The replication of S1 RNA segment in the BmCPV-infected cells was determined by RT-qPCR. (**D**) The silencing efficiencies of siRNAs to the PHB2 gene were determined by real-time PCR. BmN cells (1 × 10^6^) were transfected with 5 µmol/L of PHB2-siRNA-245, PHB2-siRNA-637 , PHB2-siRNA-750, or NC-siRNA. At 48 hpi, the cells were collected. The total RNAs extracted were used for RT-qPCR to determine the *PHB2* gene expression level. *TIF-4A* gene was used as an internal reference. (**E**) The effects of silencing PHB2 on the expression level of VP7 and active caspase3 were determined by Western blotting. BmN cells (1 × 10^6^) were transfected with 4 µg of pIZT-vsp59 plasmid for 48 h, then 5 µL of BmCPV (MOI = 2) was inoculated for 48 h, and then, respectively, transfected with PHB2-siRNA-245 at a final concentration of 5 µmol/L. Meanwhile, the inoculated cells transfected with NC-siRNA were used as a control. After 48 h, the protein was extracted and detected by Western blotting (upper). The primary antibodies were VP7 antibody (mouse, 1:1,000), active caspase3 (rabbit, 1:2,000) antibody, and α-tubulin antibody (mouse, 1:2,000), the secondary antibody is HRP-conjugated goat anti-rabbit/mouse antibody (1:5,000). The amount of protein loaded in each lane is 50 µg. Grayscale scanning analysis of Western blotting signal bands was conducted to determine the relative expression level (under). *P < 0.05, **P < 0.01, and ***P < 0.001.

The above-described observations indicated that VSP59 promoted apoptosis and arrested the cell cycle. Accordingly, we hypothesized that VSP59 might negatively regulate the viral life cycle. To examine this hypothesis, vsp59 was overexpressed in BmN cells (1 × 10^6^) with transfection of pIZT-vsp59 (4 µg). The cells transfected with pIZT-V5/His were used as a control. At 48 h post-transfection, these cells were inoculated with BmCPV (MOI = 2). At 48 hpi, the cells were collected for extraction of total RNAs and proteins to assess the BmCPV replication. Western blotting results showed that the expression levels of viral structural protein VP7 and VP4 were suppressed when vsp59 was overexpressed (Fig. 6A). Meanwhile, RT-qPCR results showed that the expression level of viral structural gene *vp1* was inhibited in cells overexpressing *vsp59* (Fig. 6B). In addition, the vsp59 mutant virus rBmCPV-VSP59null was constructed *in vitro* using a reverse genetic system to investigate the effect of VSP59 on replication of BmCPV. The results showed that compared with the recombinant BmCPV constructed by transfecting 10 RNAs transcribed from pIZT-CS1–pIZT-CS10, respectively, the replication of genomic dsRNA S1 segment was significantly inhibited in the vsp59 mutant virus rBmCPV-VSP59null (Fig. 6C). As the VSP59 had been observed as a negative regulatory factor for viral replication, it was interesting whether VSP59 interacted with PHB2 to promote apoptosis and suppress viral replication in the cells. The specific siRNAs targeting the PHB2 gene were designed, and the silencing efficiencies of these siRNAs to the PHB2 gene were determined by real-time PCR. Among the three siRNAs of PHB2, PHB2-siRNA-245 could reduce PHB2 gene expression level by 80%. Therefore, the PHB2-siRNA-245 was selected for further investigation (Fig. 6D). Here, the effect of silencing PHB2 on viral replication was assessed by Western blotting. As shown in Fig. 6E, compared with the cells treated with NC-siRNA with random sequence, intracellular viral protein VP7 increased in PHB2-siRNA-245-treated vsp59-overexpressing cells while the expression level of active caspase3 decreased. These results suggested that vsp59 promotes cell apoptosis and inhibits virus replication by interacting with PHB2.

## DISCUSSION

In this study, we describe an unrecognized sORF in the S10 dsRNA genome of BmCPV. Notably, this sORF shares the same transcript with the polyhedrin gene, as evidenced by RNA-seq data (21). Gene overlaps are a common feature of viruses, allowing them to maximize their protein repertoire within their limited genome lengths (22, 23). The proteins/peptides produced by these overlapping genes may play accessory roles in viral pathogenicity or spread (24). In this study, we identified a novel small peptide VSP59 in BmCPV virions and infected cells/midguts and found that VSP59 induces apoptosis and suppresses viral replication by targeting mitochondria. These findings enhance our understanding of the transcriptional regulation of the dsRNA genome and demonstrate the negative regulatory role of VSP59 in viral replication.

Increasing evidence suggests that alternative ORFs with biological roles are present in viruses, bacteria, and vertebrates (3, 6, 25, 26). In our previous work, we identified a potential novel viral product generated from the BmCPV segment 7 via a leaky scanning mechanism (9) and a novel small peptide with antiviral activity from the antisense strand of the S5 dsRNA segment (10). VSP59 was identified within the polyhedrin encoded by the S10 dsRNA genome of BmCPV. The sequence of this peptide is conserved among DpCPV, LdCPV, and ObCPV. Polyhedrin is a non-structural protein encoded by the S10 segment of BmCPV, and its amino acid sequences is conserved (14). Large numbers of inclusion bodies called polyhedra were produced in the cytoplasm of BmCPV-infected cells (1). Virions were embedded into the polyhedron with an ordered crystalline organization, protecting them against dehydration, freezing, and enzymatic degradation (27, 28). We investigated whether the novel conserved small peptide VSP59 encoded by S10 dsRNA genome could regulate polyhedrin expression, polyhedron assembly, or viral replication. Our findings revealed that overexpression of vsp59 suppresses the expression level of the S10 segment, even though both VSP59 and polyhedrin biosynthesis share the same S10 dsRNA template. Therefore, we hypothesize that VSP59 competes with polyhedrin for binding to S10 dsRNA, thereby negatively regulating viral proliferation.

Moreover, a signal band with 15 kDa in size, representing VSP59, was found in the BmCPV-infected cells, which was different from the theoretical molecular weight of VSP59 (59aa, about 6.5 kDa). *E. coli* lacks a post-translational modification system similar to that of eukaryotic cells (29, 30). According to the results of SDS-PAGE and Western blotting, the molecular weight of recombinant vsp59 expressed in *E. coli* was approximately 13 kDa (Fig. S2), which was more than theoretical molecular weight (6–7 kDa) of VSP59. Previous studies have indicated that the apparent molecular weights of some proteins are not exactly consistent with the theoretical molecular weight, which means that some proteins may deviate from the molecular weight determined by SDS-PAGE (31). But the molecular weight of vsp59 expressed in BmCPV-infected BmN cells or BmCPV-infected midguts was about 15 kDa, which was more than that of vsp59 expressed in *E. coli*. It may be due to the post-translational modification of vsp59 expressed in BmCPV-infected BmN cells or BmCPV-infected midguts (32–34).

Virus-derived small peptides have been found to play diverse roles in virus-host interactions. For instance, the PPxY peptide from adenoviruses can manipulate the autophagic machinery to prevent virus degradation (8). In contrast, virus-induced small peptide 1 (VISP1) encoded by cucumber mosaic virus acts as an autophagy receptor that inhibits virus infection by mediating autophagic degradation of viral suppressors of RNA silencing (12). Our previous study demonstrated that vSP27 encoded by BmCPV S5 dsRNA could inhibit virus replication (10, 13). VSP59, generated by gene overlapping, may have distinct chemical and physical properties (22). Similar to vSP27, overexpression of VSP59 suppressed viral replication (13). This result aligns with data from rBmCPV-VSP59null. Different from vSP27 encoded by circRNA (13), VSP59 is encoded by the linear transcript that is one part of S10 RNA genome. Additionally, VSP59 was identified in BmCPV virions, suggesting its potential role as an accessory peptide involved in viral replication.

During virus infection, usually, apoptosis was induced to eliminate infected cells and suppress virus propagation (35). Some viruses have evolved strategies to evade apoptosis for replication (35, 36), while others exploit it for their benefit (37–39). The regulatory mechanism of VSP59 in virus infection was different from vSP27 that activates the NF-κB signaling pathway (13). Our study revealed that VSP59 predominantly localizes to the cytoplasm and co-localizes with mitochondria, which are closely associated with apoptosis. Further investigation demonstrated that VSP59 induces apoptosis in a dose-dependent manner. Notably, VSP59 was found in BmCPV virions, indicating its potential role in inducing apoptosis during the early stages of viral infection. Our findings suggest that VSP59 interacts with an inner membrane mitophagy receptor, PHB2 (40). PHB2 is known to regulate mitochondrial membrane protein degradation by modulating mitochondrial proteases involved in mitophagy (40). Silencing the *PHB2* gene weakened the inhibitory effect of VSP59 on BmCPV replication.

In conclusion, we have found a small peptide VSP59, which is encoded by sORF in S10 dsRNA genome of BmCPV. The VSP59 could induce apoptosis and inhibit virus replication by interacting with PHB2. The mechanism might be that interaction of VSP59 with PHB2 active mitochondrial depolarization and proteasome-dependent outer membrane rupture, which could recruit LC3 (autophagosomal membrane-associated protein) to PHB2 or recruit PARL to induce PINK1-PRKN/Parkin-dependent mitophagy (41, 42). However, our study provides insights into the role of VSP59 in BmCPV replication, but several limitations remain. The exact roles of VSP59 in the assembly of polyhedra and the association between mitophagy and VSP59 require further investigation. Additionally, the biogenesis of vsp59 needs to be explored.

## SUPPLEMENTAL MATERIAL

Supplemental material spectrum.00826-24-s0001.docx Fig. S1 to S5.

ASM does not own the copyrights to Supplemental Material that may be linked to, or accessed through, an article. The authors have granted ASM a non-exclusive, world-wide license to publish the Supplemental Material files. Please contact the corresponding author directly for reuse.
