# Supplementary material for: Identification and characterization of a novel small viral peptide (VSP59) encoded by Bombyx mori cypovirus (BmCPV) that negatively regulates viral replication
Source: Microbiol Spectr. 2024 Oct 9;12(11):e00826-24. doi: 10.1128/spectrum.00826-24 (PMC11537000; doi:10.1128/spectrum.00826-24)
Supplement: Supplemental material — Fig. S1 to S5. [file spectrum.00826-24-s0001.docx]

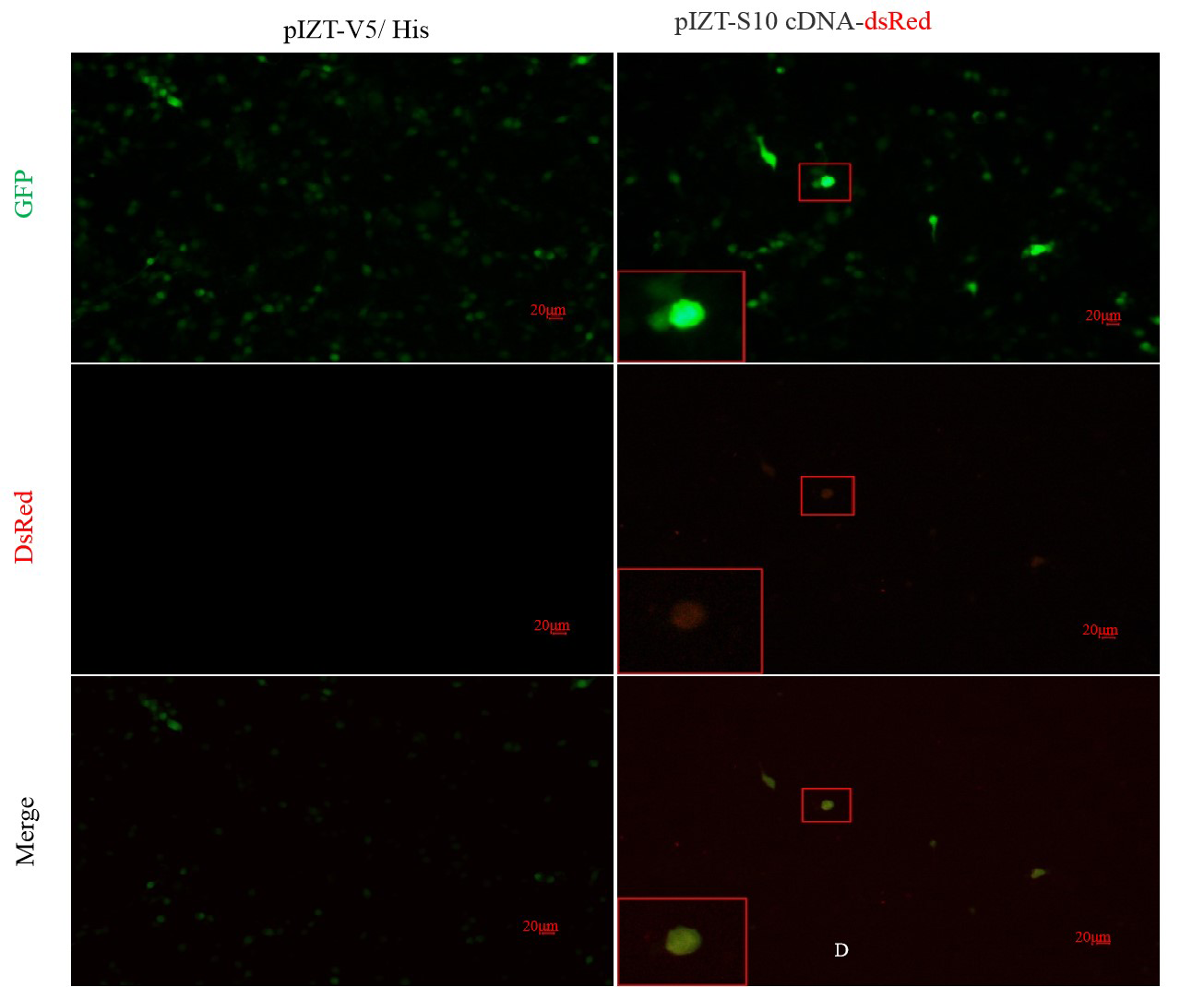


Supplementary Figure 1 The expression of dsRed was visualized. BmN cells (1×10^4^) were transfected with 0.5 µg of pIZT-V5/His and pIZT-V5/His-S10 cDNA-dsRed, respectively. After 48 h, red fluorescence was observed in some cells transfected with pIZT-V5/His-S10 cDNA-dsRed.


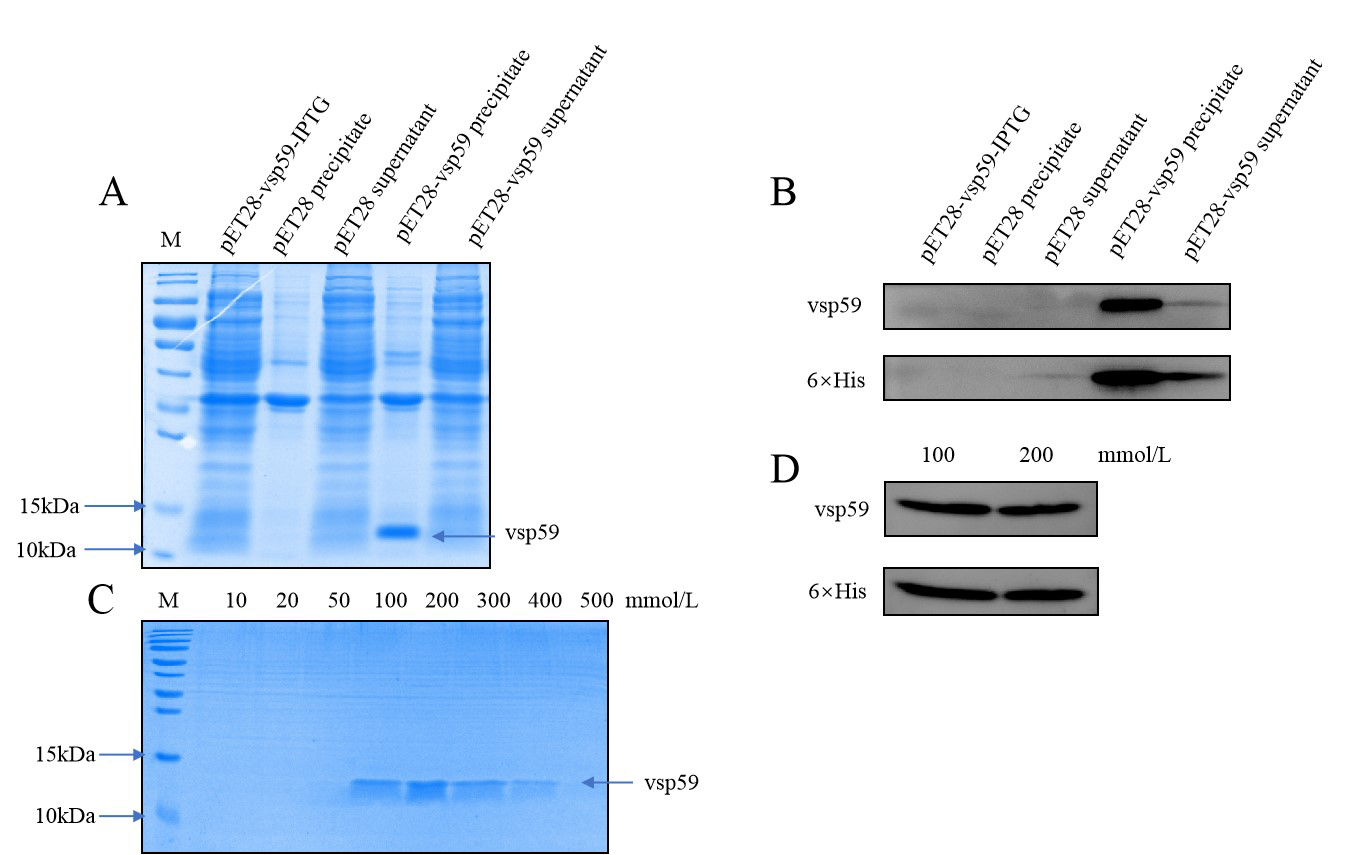


Supplementary Figure 2 Expression of VSP59 in transformed *E. coli* with pET28-vsp59 and preparation of anti-VSP59 antibody. (A) SDS-PAGE detection of recombinant VSP59 expressed in transformed *E. coli* with pET28-vsp59. pET28-vsp59-IPTG: pET28-vsp59 transformed bacteria without induction by IPTG; pET28 precipitate: The precipitate of pET-28a (+) transformed bacteria induced by IPTG after ultrasonic treatment; pET28 supernatant: The supernatant of pET-28a (+) transformed bacteria induced by IPTG after ultrasonic treatment; pET28-vsp59 precipitate: The precipitate of pET28-vsp59 transformed bacteria induced by IPTG after ultrasonic treatment; pET28-vsp59 supernatant: The supernatant of pET28-vsp59 transformed bacteria induced by IPTG after ultrasonic treatment. (B) Western blotting detection of recombinant VSP59. The samples for each lane were consistent with those shown in (A). The primary antibody were vsp59 antibody (mouse, 1:1000) and a 6×His antibody (mouse, 1:2000), and the secondary antibody was HRP conjugated goat anti-mouse IgG (1:5000). (C)SDS-PAGE detection of recombinant protein VSP59 purified by gradient elution with imidazole at different concentrations of 10, 20, 50, 100, 200, 300, 400 and 500 mmol/L. (D)Western blotting detects of recombinant protein VSP59 purified by gradient elution with imidazole at 100 and 200 mmol/L, respectively. The primary antibody was vsp59 antibody (mouse, 1:1000) and 6 × His antibody (mouse, 1:2000), and the secondary antibody was HRP conjugated goat anti-mouse IgG (1:5000).


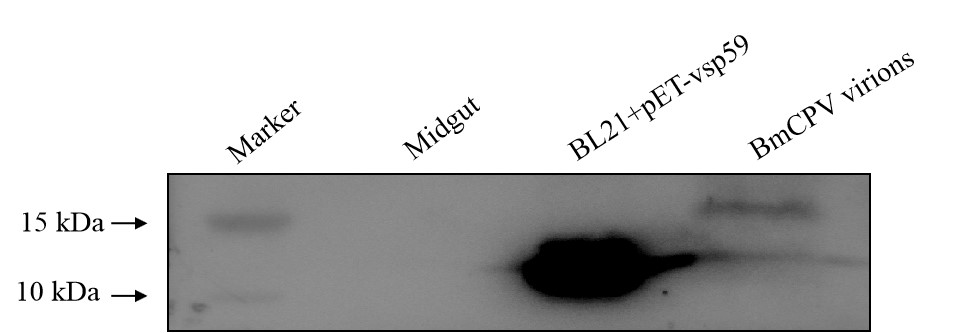


Supplementary Figure 3 Identification of VSP59 from the BmCPV virions. The purified polyhedra were lysed with lysis buffer (pH>10.5), and the virions released from the polyhedra were used for Western blotting to detect VSP59.Midgut: midgut protein of healthy silkworm (negative control); BL21+PET-vsp59: recombinant vsp59 expressed in *E. coli* (positive control); BmCPV virions: the virions released from the polyhedra; Marker, protein marker. The primary antibody were vsp59 antibody (mouse, 1:1000), and the secondary antibody was HRP conjugated goat anti-mouse IgG (1:5000). The amount of protein loaded in each lane was 40 µg.


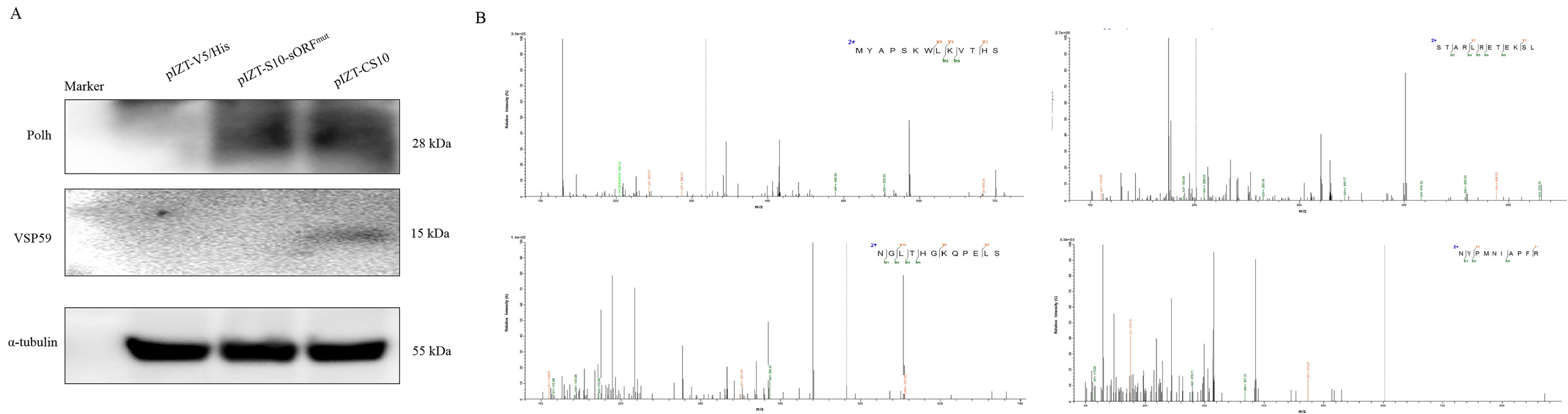


Supplementary Figure 4 Identification of VSP59 in BmCPV-infected midguts. (A) VSP59 was encoded by the BmCPV genomic RNA S10 segment. 4μg of pIZT-V5/His, pIZT-CS10 and pIZT-S10-sORF^mut^ plasmids were transfected into BmN cells (1×10^6^), respectively. The total proteins extracted from the transfected cells at 48 h post-transfection were used for SDS-PAGE and Western blotting with specific anti-Polh, anti-VSP59 antibodies. (B) Mass spectrometry identification of VSP59.


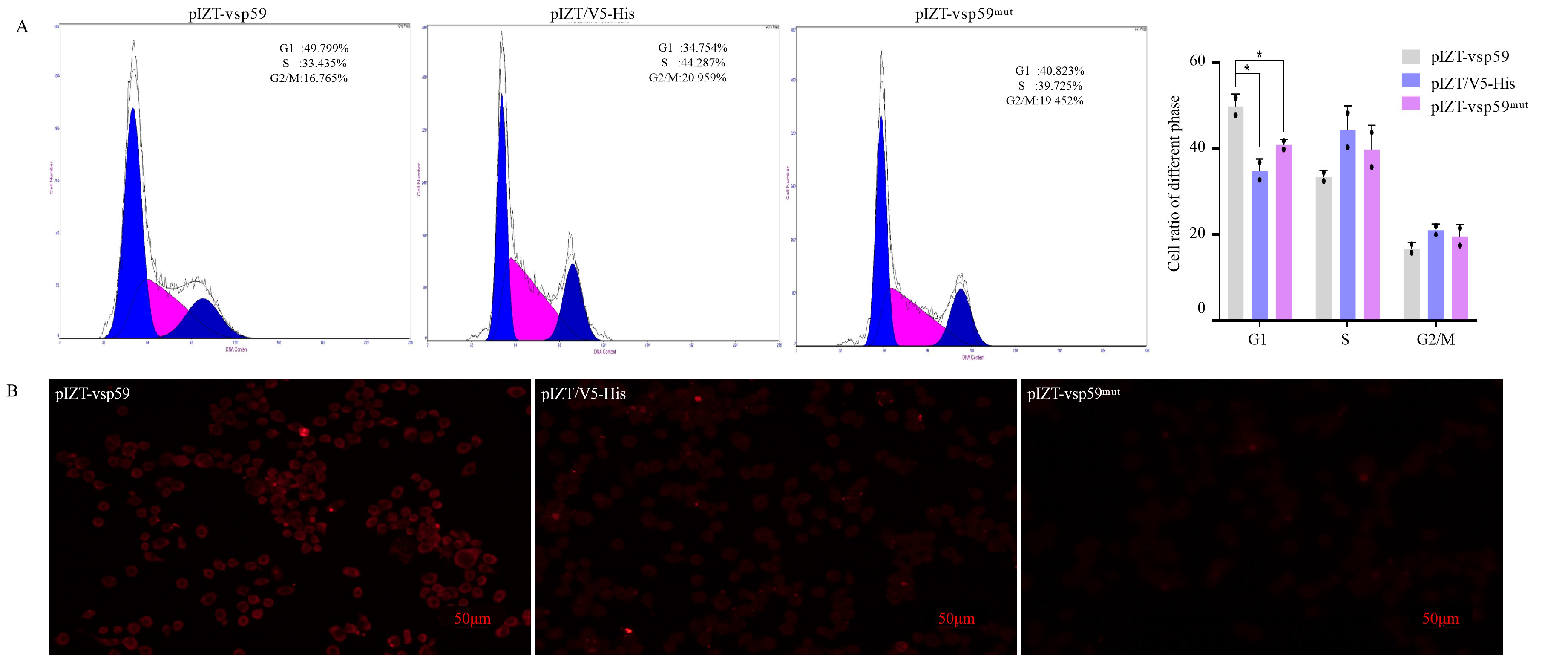


Supplementary Figure 5 VSP59 arrests the cell phase and induces apoptosis. (A) VSP59 arrests the cell phase. BmN cells (1×10^6^) were transfected with 4µg of pIZT-V5/His (a negative control), pIZT-vsp59, and pIZT-vsp59^mut^plasmids. At 48 h post-transfection, the cell phase was detected by flow cytometry analysis. (B) Apoptosis was induced by overexpression of vsp59. BmN cells (1 × 1 0^4^) were transfected with 1 µg of pIZT-vsp59, pIZT-V5/ His and pIZT-vsp59^mut^, respectively. After 48 hours, the cells were stained with TUNEL kit (red) and observed under fluorescence microscope. *, p<0.05.
